# Mechanisms of transcranial direct current stimulation (tDCS) for pain in patients with fibromyalgia syndrome

**DOI:** 10.3389/fnmol.2024.1269636

**Published:** 2024-01-30

**Authors:** Shan Wang, Shu-Hao Du, Xue-Qiang Wang, Jun-Yan Lu

**Affiliations:** ^1^Department of Health School, Shanghai Normal University Tianhua College, Shanghai, China; ^2^Department of Sport Rehabilitation, Shanghai University of Sport, Shanghai, China; ^3^Rehabilitation Medicine Center, The Second Affiliated Hospital of Wenzhou Medical University, Wenzhou, Zhejiang, China; ^4^School of Rehabilitation Medicine, Wenzhou Medical University, Wenzhou, Zhejiang, China; ^5^Department of Rehabilitation Medicine, Shanghai University of Medicine and Health Sciences Affiliated Zhoupu Hospital, Shanghai, China

**Keywords:** chronic pain, mechanisms, brain modulation, tDCS, fibromyalgia syndrome

## Abstract

Fibromyalgia syndrome (FMS) is a recurrent pain condition that can be challenging to treat. Transcranial direct current stimulation (tDCS) has become a promising non-invasive therapeutic option in alleviating FMS pain, but the mechanisms underlying its effectiveness are not yet fully understood. In this article, we discuss the most current research investigating the analgesic effects of tDCS on FMS and discuss the potential mechanisms. TDCS may exert its analgesic effects by influencing neuronal activity in the brain, altering cortical excitability, changing regional cerebral blood flow, modulating neurotransmission and neuroinflammation, and inducing neuroplasticity. Overall, evidence points to tDCS as a potentially safe and efficient pain relief choice for FMS by multiple underlying mechanisms. This article provides a thorough overview of our ongoing knowledge regarding the mechanisms underlying tDCS and emphasizes the possibility of further studies to improve the clinical utility of tDCS as a pain management tool.

## 1 Introduction

Fibromyalgia syndrome (FMS) is a chronic disorder characterized by widespread musculoskeletal pain and tenderness in at least 11 areas for over 3 months (Galvez-Sánchez and Reyes Del Paso, 2020). Persistent musculoskeletal pain was linked to worse physical and cognitive function, burdening individuals and society (Xu et al., 2023; Zheng et al., 2023). Along with musculoskeletal pain, people with FMS often report fatigue, dyscognition, stiffness, sleep disturbances, mood issues, and hypervigilance, further reducing quality of life (Arnold et al., 2019; Gyorfi et al., 2022). FMS affects 2–4% of people worldwide (Häuser and Fitzcharles, 2018), with 7% of women aged 50–80 affected (White and Robinson, 2015). Despite improvements in FMS therapy, managing pain remains difficult for healthcare providers. Brain stimulation has been shown to alleviate FMS in recent clinical trials (Hou et al., 2016), giving hope for people with FMS.

Transcranial direct current stimulation (tDCS), a typical non-invasive brain stimulation technique, is being considered as an FMS treatment (Teixeira et al., 2022). It can change the polarity-dependent excitability of the cerebral cortex by delivering a low electrical current to specific brain areas via two electrodes on the scalp (Vicario et al., 2020). Anodal tDCS usually depolarizes and excites the neuronal membrane potential, while cathodal tDCS does the opposite (Sehm et al., 2013; Ho et al., 2016). Compared to other brain stimulation methods, TDCS is non-invasive, inexpensive, and safe (Mosayebi Samani et al., 2019).

Current research on tDCS for FMS is promising, and it has been recommended by the European Chapter of the International Federation of Clinical Neurophysiology as a possible effective treatment for FMS (Level B) (Lefaucheur et al., 2017). Despite multiple clinic studies (Caumo et al., 2022; Ramasawmy et al., 2022) and systematic reviews (Lloyd et al., 2020; Teixeira et al., 2022) showing that tDCS reduces FMS pain, a specific research gap remains. Initial results, like pain alleviation, are the focus of these investigations. The complicated pathophysiological changes in FMS pain and the underlying mechanisms by which tDCS relieves FMS pain are unknown. Thus, a deeper understanding of the mechanism of tDCS in FMS is required to enhance its validity and repeatability (van Boekholdt et al., 2021). Moreover, tDCS treatment parameters in FMS vary greatly across different studies. Some studies (Matias et al., 2022; Ramasawmy et al., 2022) recommend stimulating the left primary motor cortex (M1), while others (Forogh et al., 2021; Caumo et al., 2022) propose the dorsolateral prefrontal cortex (DLPFC) as more efficacious. Key treatment parameters like stimulation duration, intensity, and frequency are also inconsistently described and used. Lack of consistency makes clinical use of tDCS for FMS difficult. Thus, our work aims to (1) improve understanding of FMS’s complex pathophysiological changes and the mechanisms by which tDCS reduces pain; and (2) evaluate the effects and treatment parameters of tDCS on FMS.

## 2 Effect of tDCS on pain for fibromyalgia

Many studies have aimed to enhance the impact of tDCS stimulation on pain symptoms in fibromyalgia patients. However, the complexity and heterogeneity across these studies prompted us to conduct a scoping review following established guidelines, including the Preferred Reporting Items for Systematic Reviews and Meta-Analyses (PRISMA-SR) Statement, to provide a concise and efficient summary of the existing literature (Peters et al., 2015; Tricco et al., 2018). Eligibility criteria were developed using the SPIDER approach (Cooke et al., 2012).

### 2.1 Specify sample

The review included patients diagnosed with fibromyalgia by local rheumatology associations or other formal institutions. Most studies excluded individuals receiving additional medication to prevent a potential impact on trial results. To ensure study homogeneity, female subjects were predominantly included, given the higher prevalence of fibromyalgia in women. Additionally, certain studies specified a minimum 6-month duration of chronic pain among participants to investigate its effects on individuals with prolonged pain experiences.

### 2.2 Phenomenon of interest

Most studies aimed to explore the impact of tDCS stimulation on pain, disability, and quality of life in fibromyalgia patients. The primary brain regions stimulated were M1 and DLPFC, typically with an intensity of 1–2 mA and a duration of 20 min. Randomized controlled trials commonly employed sham tDCS as controls, while only one study compared the effects of repetitive transcranial magnetic stimulation (rTMS) and tDCS on pain and quality of life in fibromyalgia patients (Forogh et al., 2021).

### 2.3 Design of the study

Most of the studies were randomized controlled trials, with five studies using a double-blind approach (Riberto et al., 2011; Villamar et al., 2013; Khedr et al., 2017; Caumo et al., 2022; Ramasawmy et al., 2022), two studies using a cross-over design (Valle et al., 2009; Villamar et al., 2013), and two studies exploring the long-term efficacy of tDCS (Cummiford et al., 2016; Silva et al., 2017).

### 2.4 Evaluation

This study primarily investigated the impact of tDCS stimulation on pain relief in fibromyalgia patients, focusing on pain outcome measures. Visual Analogue Scale (VAS), Numeric Rating Scale (NRS), and Pain Pressure Threshold (PPT) were chosen as the primary pain indicators. Most studies observed that applying 2 mA anodic tDCS to the M1 and DLPFC regions effectively alleviated pain (Roizenblatt et al., 2007; Valle et al., 2009; Villamar et al., 2013; Cummiford et al., 2016; Khedr et al., 2017; Silva et al., 2017; Kang et al., 2020; Forogh et al., 2021; Caumo et al., 2022).

### 2.5 Research type

The majority of literature in this study adopts quantitative research methods, primarily comparing the impacts of genuine and sham tDCS stimulation on pain and other functions in patients. While some studies reported no significant changes in pain with sham tDCS, one interesting finding contradicted this trend, suggesting that sham tDCS could exhibit similar analgesic effects, possibly linked to the placebo analgesic effect (Caumo et al., 2022).

**FIGURE 1:**
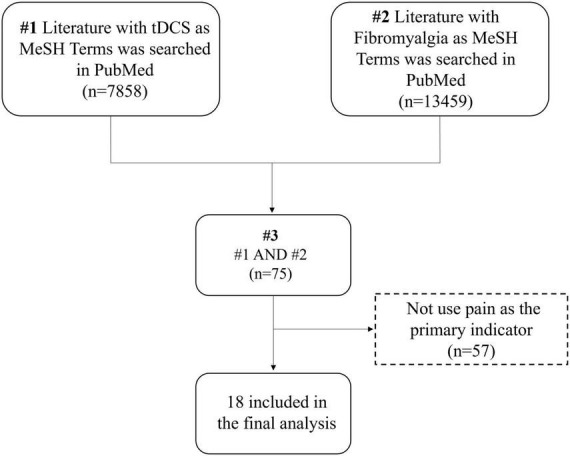
Flow chart for researches enrolled in this study.

“Transcranial Direct Current Stimulation” and “Fibromyalgia” served as MeSH Terms. On November 7, 2022, 75 pertinent studies were retrieved from the PubMed database, underwent manual screening, and ultimately, 18 relevant studies were included (Figure 1). The detailed search strategy is available in the Supplementary material. Research indicates that 10 sessions of anodal tDCS in the M1 region can decrease pain levels in fibromyalgia patients. The development of this condition may be linked to alterations in serum endorphin levels (Khedr et al., 2017). Whether applied singly or periodically, tDCS mitigates pain perception, and stimulating the DLPFC region proves beneficial for relieving fatigue (To et al., 2017). A single 2 mA, 20-min session of tDCS stimulation in the M1 and Supra-orbital area (SO) can yield positive clinical effects (To et al., 2017). Moreover, limited research has addressed enhancing functional connectivity in pain-related brain regions through tDCS. Future studies should employ multiple imaging techniques to observe changes in the brain mechanisms of tDCS analgesia (Caumo et al., 2022). Table 1 provides details on tDCS stimulus parameters and the results of the included studies.

**TABLE 1:** Characteristics of studies included in the review.

				Stimulation details					
**References**	**Participants(n)**	**Study aim**	**Study design**	**Site**	**Control group**	**Current (mA)**	**Duration** **Frequency Sessions**	**Pain Outcome**	**Result**
Caumo et al., 2022	Female fibromyalgia (*n* = 48)	To explore the effect of two-frontal home tDCS on pain disaster and disability in fibromyalgia	Randomized, double-blind sham-controlled trial	L-DLPFC A	s-tDCS	2	20 min Daily 20	PCS; HPTo	A-tDCS reduced PCS by 51.38% and increased HPTo
Cummiford et al., 2016	Female fibromyalgia (*n* = 12)	To investigate how a clinically relevant schedule of tDCS sessions alters resting state FC and how these changes might relate to clinical pain	Crossover design trial	M1 A	s-tDCS	2	20 min Daily 5	VAS	Clinical pain significantly decreased (*p* = 0.038)
De Ridder and Vanneste, 2017	Fibromyalgia (*n* = 19); healthy control (*n* = 19)	To unravel the neural mechanisms involved in global pain suppression, mediated by occipital nerve field stimulation, within the realm of fibromyalgia	Controlled trial	OCF C	s-tDCS; healthy control	1.5	20 min Daily 7	NRS; PCS	A significant effect in NRS (*F* = 23.14, *p* < 0.001) and PCS (*F* = 19.17, *p* < 0.001)
Fagerlund et al., 2015	Fibromyalgia (*n* = 48)	To test the effect of tDCS stimulation on pain in patients with fibromyalgia	Randomized controlled trial	M1 A	s-tDCS	2	20 min Daily 5	NRS	No significant differences between two groups
Forogh et al., 2021	Fibromyalgia (*n* = 30)	To compare the rTMS and tDCS on pain and quality of life in patients with fibromyalgia	Randomized controlled trial	DLPFC A	rTMS	2	20 min Daily 7	VAS	26.6% of patients in tDCS group experienced at least a 30% reduction of VAS from baseline to last follow-up (*p* = 0.028)
Kang et al., 2020	Fibromyalgia (*n* = 46)	To explored the efficacy, tolerability, and safety of tDCS treatment in patients with fibromyalgia	Intervention and follow-up trial	M1 A/C	/	2	20 min Daily 5	VAS; BPI	A significant decrease and improvement in VAS (*p* < 0.001) and BPI (*p* < 0.01)
Khedr et al., 2017	Fibromyalgia (*n* = 40)	To evaluate the effects of tDCS in relieving fibromyalgia pain and its relation with beta-endorphin changes	Double blinded, randomized clinical trial	L-M1 A	s-tDCS	2	20 min Daily 10	WPI; VAS	A significant improvement on the a- tDCS group in WPI and VAS (*P* = 0.001)
Matias et al., 2022	Female fibromyalgia (*n* = 31)	To investigate the effects of tDCS associated with functional exercise on pain, functional performance, psychological symptoms, and quality of life of patients with fibromyalgia	Randomized controlled trial	M1 A	s-tDCS + functional exercises	2	20 min Daily 5	VAS; PPT	No significant differences between two groups (*P* > 0.05)
Mendonca et al., 2011	Fibromyalgia (*n* = 30)	To determine current distribution and short-term analgesic effects of tDCS in fibromyalgia using different electrode montages	Randomized controlled trial	M1 A/C SO A/C	s-tDCS	2	20 min Single 1	VNS; PPT	A significant improvement on the SO in pain
Mendonca et al., 2016	Fibromyalgia (*n* = 45)	To assess whether the combined intervention of tDCS and aerobic exercise would induce significantly greater pain reduction as compared to tDCS alone and aerobic exercise alone	Randomized placebo-controlled clinical trial	L-M1 A R- SO C	tDCS; AE	2	20 min Daily 10	VNS; PPT	A significant improvement in VNS and PPT (*p* < 0.001)
Ramasawmy et al., 2022	Fibromyalgia (*n* = 30)	To investigate the preliminary clinical efficacy and feasibility of combining MM and Tdcs for pain and associated symptoms in patients with fibromyalgia syndrome	Randomized, double-blind sham-controlled trial	L-M1 A	s-tDCS + MM	2	20 min Daily 10	NRS; PPT	No significant differences between two groups
Riberto et al., 2011	Female fibromyalgia (*n* = 23)	To test whether active tDCS, as compared with sham tDCS, combined with multidisciplinary rehabilitation is associated with significant clinical gains in fibromyalgia	Randomized, double-blinded controlled trial	M1 A SO C	s-tDCS	2	20 min Daily 10	VAS; PPT	No significant differences between two groups
Roizenblatt et al., 2007	Fibromyalgia (*n* = 32)	To investigate whether active tDCS of DLPFC and M1 as compared to sham treatment is associated with changes in sleep structure in fibromyalgia	Randomized, sham-controlled trial	M1/DLPFC A	s-tDCS	2	20 min Daily 5	VAS	59.14% decrease in M1 site in VAS
Silva et al., 2017	Female fibromyalgia (*n* = 40)	To test the effects of a single session of tDCS coupled with a Go/No-go task in modulating three distinct attentional networks	Randomized controlled trial	DLPFC A	s-tDCS	1	20 min Single 1	HPT; HPTo	A- tDCS significantly increased the HPT (*P* < 0.001) and HPTo
To et al., 2017	Fibromyalgia (*n* = 42)	To explore the effectiveness of repeated sessions of tDCS (eight sessions) targeting the C2 area and DLPFC in reducing fibromyalgia symptoms, more specifically pain and fatigue	Randomized controlled trial	DLPFC/C2 A/C	s-tDCS	1.5	20 min Daily 8	NRS; PCS	C2 and DLPFC tDCS significantly improved pain (*P* < 0.001)
Valle et al., 2009	Female fibromyalgia (*n* = 41)	To determine whether a longer treatment protocol tDCS of the M1 or DLPFC could offer additional, more long-lasting clinical benefits in the management of pain from fibromyalgia	Randomized, sham-controlled longitudinal clinical trial	DLPFC/L M1 A	s-tDCS	2	20 min Daily 10	VAS	M1 tDCS significantly improved pain (*p* = 0.011)
Villamar et al., 2013	Fibromyalgia (*n* = 18)	To examine the effects of a novel, more focal method of tDCS on overall perceived pain in fibromyalgia patients	Double blinded, sham-controlled, crossover trial	L-M1 A/C	s-HD-tDCS	2	20 min Single 1	VNS; PPT	A significant improvement in pain (*p* = 0.004)
Yoo et al., 2018	Fibromyalgia (*n* = 58)	To test the effect of combining 2 targets of stimulation using tDCS	Randomized controlled trial	DLPFC A/C ON A/C	s-tDCS	2 (DLPFC) 1.5 (ON)	20 min Daily 8	NRS	No significant differences between two groups

s-tDCS, shame transcranial direct current stimulation; l-DLPFC, left dorsolateral prefrontal cortex; PCS, Pain Catastrophizing Scale; HPT, heat pain threshold; Hpto, heat pain tolerance; M1, primary motor cortex; A, anodal stimulation; C, cathodal stimulation; s, sham stimulation; R, right; VAS, visual analogue scale; NRS, numeric rating scale; OCF, Occipital nerve field; rTMS, repetitive transcranial magnetic stimulation; BPI, Brief Pain Inventory; WPI, widespread pain index; PPT, Pain pressure threshold; SO, supra-orbital area; VNS, visual numerical scale; AE, aerobic exercise; MM, mindfulness meditation; HD-tDCS, high-definition transcranial direct current stimulation; ON, occipital nerve.

## 3 Mechanisms of tDCS for fibromyalgia syndrome

**FIGURE 2:**
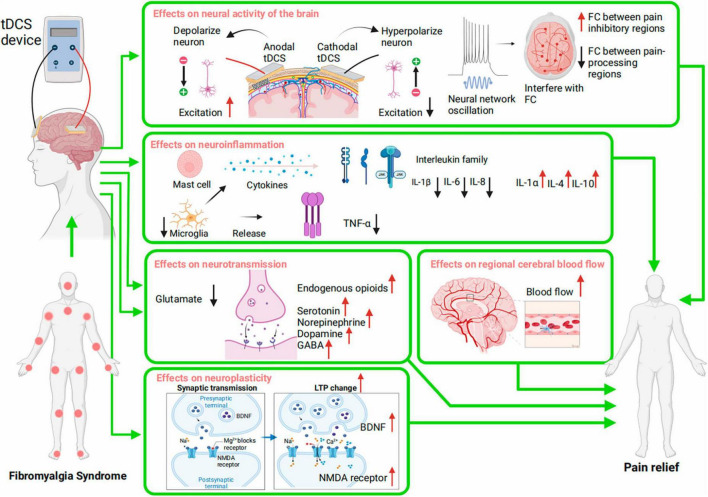
Potential Mechanisms of tDCS for pain in Fibromyalgia Syndrome (FMS). The mechanisms include regulation of neural activity; modulation of neuroinflammation; regulation of neurotransmission; regulation of regional cerebral blood flow; modulation of neuroplasticity. FC, function connectivity; TNF, tumor necrosis factor; IL, interleukin; GABA, gamma-aminobutyric acid; LTP, long-term potentiation; BDNF, brain-derived neurotrophic factor; NMDA receptors, N-methyl-D-aspartate receptors.

Despite the unknown pathophysiology of FMS, pain is connected to central sensitization (Rehm et al., 2021), a heightened sensitivity of the nervous system that overreacts to stimuli. This process is essential in FMS, causing widespread pain and other sensory-related symptoms. Abnormal brain neural networks and excitability, neuroinflammatory processes, neurotransmitter imbalances, abnormal cerebral blood flow, and disrupted neuroplasticity in the pain-processing region may be involved in this process and contribute to FMS pain (Gyorfi et al., 2022). Although tDCS has shown promise in reducing FMS pain, its specific mechanism is unknown, and no biomarkers are available to predict a patient’s response. The consensus is that tDCS depolarizes or hyperpolarizes neuronal membrane potential, affecting neural excitability (Lefaucheur and Wendling, 2019). This knowledge allows for further study of its mechanics. Multiple systems may be involved in how tDCS reduces FMS pain. Possible mechanisms are the promotion of cortical excitability recovery as well as effects on neuroinflammation, neurotransmission, regional cerebral blood flow (rCBF), and neuroplasticity. The mechanism of tDCS on FMS pain is depicted in Figure 2.

### 3.1 Effects on neural activities of the brain

With aberrant central nervous system excitability (Thabit et al., 2021), pain-processing brain regions in FMS sufferers are more hypersensitive to pressure stimuli than healthy people (Truini et al., 2015).

An abnormal mix of enhanced and reduced Functional Connectivity (FC) patterns across the pain matrix was found in individuals with FMS (Cifre et al., 2012), indicating the neural networks involved in pain perception and processing are functioning abnormally.

Anodal tDCS increases while cathodal decreases neural excitability in targeted areas (Pellicciari et al., 2013). A study (Auvichayapat et al., 2018) found that anodal tDCS on M1 increased neuronal activity in that area and decreased neuropathic SCI pain, implying that pain relief occurs by increasing M1 excitability, which is related to pain process (Zortea et al., 2019). Another study discovered that anodal tDCS targeting DLPFC increased DLPFC neural excitability, emotion, and pain relief in participants (Maeoka et al., 2012). Since the DLPFC regulates emotional pain perception, the change may affect how people perceive painful sensations. These synchronous changes suggest tDCS may be helpful to regulate abnormal pain processing and perception in FMS.

Patients with FMS displayed decreased FC between key pain-modulating regions (Jensen et al., 2012) and altered FC among pain process regions and sensorimotor areas (Flodin et al., 2014) relative to healthy individuals. Pain intensity correlates with FC sensory integration disturbances (Pujol et al., 2014). Polanía et al. (2012) found that anodal tDCS on M1 improved the FC between the left thalamus and ipsilateral M1 in healthy persons. Cummiford et al. (2016) found that anodal tDCS on the left M1 and cathodal tDCS on the right supraorbital cortex in FMS sufferers reduced FC between the left ventral posterolateral thalamus seed and left inferior parietal lobule, and FC between periaqueductal gray seed and posterior cingulate, followed by decreased pain. TDCS appears able to modulate disrupted FC in FMS, which may underlie its pain-relieving effects in these patients.

Moreover, tDCS can alter oscillatory activity of brain at a network level (Donaldson et al., 2019). These oscillations integrate and separate brain areas involved in sensory-painful perception and processing (Kim and Davis, 2021). Research found enhanced oscillations in the anterior cingulate and prefrontal cortex of FMS patients correlated with increased pain, fatigue, and stress during rest (Fallon et al., 2018). Another study demonstrated that a high-definition tDCS modulated oscillations and reduced FMS pain (Castillo-Saavedra et al., 2016). These alterations suggest that tDCS may reduce pain by modulating abnormal neural oscillations in FMS.

In all, tDCS may relieve FMS pain by modulating cortical excitability, FC, and neural oscillations.

### 3.2 Effects on neuroinflammation

Neuroinflammation refers to inflammatory processes within the central nervous system that are known to exacerbate pain sensations in FMS (Mendieta et al., 2016). An imbalance between pro- and anti-inflammatory cytokines in cerebrospinal fluid (CSF) is common in FMS. Studies showed increased pro-inflammatory chemokines/cytokines interleukin 1 (IL-1), IL-6, IL-8, and TNF-α, and decreased anti-inflammatory cytokines IL-4 and IL-10 in the CSF of FMS sufferers compared to healthy individuals (Ross et al., 2010; Mendieta et al., 2016). Moreover, microglia and mast cells (MCs) are engaged in FMS, activated to secrete more pro-inflammatory Cytokines (Theoharides et al., 2019). Pro-inflammatory cytokine dysregulation aggravates low-grade inflammation in CNS, activates or even sensitizes nociceptors, causes pain sensitization, and triggers hyperalgesia (Siracusa et al., 2021).

Transcranial direct current stimulation may reduce FMS pain by modulating neuroinflammation, possibly achieved by stimulating brain immune cells, such as MCs and glial cells, to regulate pro-inflammatory cytokines release. Research showed tDCS can reduce the activation of microglia (Walter et al., 2022), a type of essential glial cell in the neuroinflammatory process, thus decreasing the synthesis of TNF and other inflammatory mediators (Guo et al., 2020).

Animal models show that tDCS can change neuroinflammatory mediators. IL-1β (Lopes et al., 2020; Regner et al., 2020) and IL-6 (Guo et al., 2020) were reduced in the CNS structure of animals following tDCS stimulation, while IL-1α (Santos et al., 2020), IL-10 (Santos et al., 2020), and IL-4 (Lopes et al., 2019) were increased. Moreover, animals in these experiments showed analgesic response after tDCS stimulation, which provided a window into the pain relief caused by neuroinflammatory modulation. Human studies also confirmed the analgesic effect of tDCS caused by the regulation of neuroinflammation. A sham-controlled study found plasma IL-8 reduced significantly among bipolar disorder sufferers after using tDCS (Goerigk et al., 2021). In other studies, depressed individuals had a non-significant decrease in plasma IL-6 and TNF-α compared to the sham group after tDCS activation (Brunoni et al., 2014, 2018). These findings suggest tDCS may relieve FMS pain by modulating neuroinflammation through balancing pro- and anti-inflammatory cytokines. Further tDCS studies in FMS patients with a focus on cytokines are needed to confirm the consistency of the changed cytokine and analgesic response, verifying its ability to influence neuroinflammation for pain relief.

### 3.3 Effects on neurotransmission

Pain in FMS may be associated with an impairment of excitatory and inhibitory neurotransmission (Harris, 2010). Abnormal levels of neurotransmitters were found in the CSF and brain of FMS patients, such as glutamate and substance P, serotonin (5-HT), noradrenaline, dopamine, and gamma-aminobutyric acid (GABA) (Clauw et al., 2011). Changed neurotransmitter levels increased pro-nociceptive transmission and reduced anti-nociceptive transmission. Changed endogenous cerebral opioid activation is another anomaly in FMS (Schrepf et al., 2016).

Transcranial direct current stimulation shows promise for reducing FMS pain by regulating neurotransmitters implicated in its complex pathophysiology. Increased levels of glutamate (excitatory) and reduced levels of GABA (inhibitory) contribute to FMS hyperalgesia (Harris, 2010; Pomares et al., 2020). Studies (Zhao et al., 2020; Lengu et al., 2021) show that tDCS can modulate cortical levels of GABA and glutamate, impacting neuronal signaling. Bifrontal tDCS (anode over left DLPFC and cathode over right DLPFC with a current of 2 mA) increased dopamine in the ventral striatum in healthy participants (Fonteneau et al., 2018). Research found that tDCS with an anode on the left and a cathode on the right DLPFC in healthy subjects enhanced left striatal GABA, correlated with increased right striatal dopamine, and decreased GABA in the left DLPFC (Bunai et al., 2021). Additional research shows tDCS can also affect serotonin (Brunoni et al., 2013) and noradrenaline (Mishima et al., 2019) release. Changes in transmitters induced by tDCS may activate pain inhibitory pathways to cause pain relief in FMS.

Deficiencies in an endogenous pain management system may induce widespread pain in FMS (Schrepf et al., 2016). The intrinsic pain-regulating system modulates spinal cord pain signals via the descending brainstem-to-spinal cord pathway. This system appears to be strengthened by TDCS to reduce pain signaling and thus relieve pain (DosSantos et al., 2018). Research also linked pain relief to increased beta-endorphin levels (Chaudhry and Gossman, 2021). A review found that tDCS enhances dysfunctional neuronal circuitries involved in the pain-descending inhibitory system associated with opioids, thereby reducing chronic non-cancer-related pain (Zortea et al., 2019). DosSantos et al. (2012) discovered that tDCS over M1 boosted the endogenous-opioid release and the experimental cold pain threshold in a subject with trigeminal neuropathic pain. Another clinical trial (Khedr et al., 2017) indicated that left M1 tDCS reduced pain, improved mood, and boosted β-endorphin levels in FMS sufferers. To summarize, tDCS affects glutamate, serotonin, noradrenaline, dopamine, GABA, and endogenous brain opioids. These modulations may explain tDCS’ analgesic impact.

### 3.4 Effects on regional cerebral blood flow

People with FMS suffer abnormal rCBF and metabolism in pain-related regions, which may contribute to pain severity. Patients with FMS have lower CBF than controls in different brain regions, including the thalamus, caudate nucleus, pontine tegmentum, and basal ganglia (Kwiatek et al., 2000; Schmidt-Wilcke et al., 2007; Shokouhi et al., 2016). Given that these areas play a crucial role in processing and regulating pain, the reduction in CBF may be a major consideration in the heightened sensitivity to pain and chronic discomfort suffered by patients with FMS. Alterations in metabolism were also found in different regions of the brain in individuals with FMS (Guedj et al., 2008), which were related to how well the disorder would progress (Usui et al., 2017).

A study (Zheng et al., 2011) showed that anodal tDCS significantly raised rCBF (17.1%) during stimulation, which returned to baseline afterward, while cathodal tDCS caused a smaller rCBF increase in participants. La Rocca et al. (2022) found TDCS stimulation on M1 restored basic cortical hypometabolism in patients with FMS. Jales Junior et al. (2015) found that tDCS significantly increased rCBF in basal ganglia, and this alteration correlates with reduced pain in patients with FMS. These regions are critical to pain processing. Negative rCBF and cortical hypometabolism can affect neuronal function and pain processing. These studies collectively suggest that tDCS modulates rCBF and hypometabolism, which may normalize the dysfunctional neural circuits involved in pain perception, thereby reducing the pain experienced by FMS patients.

### 3.5 Effects on neuroplasticity

Transcranial direct current stimulation may reduce FMS pain by altering the brain’s pain response by inducing plasticity. Neuroplastic changes, including long-term potentiation (LTP) and long-term depression (LTD) (Kourosh-Arami et al., 2021), refer to the ability of the brain to reshape itself by generating new neural connections. Because of this adaptability, FMS causes an overactive brain pain processing system and generalized widespread pain (Gerra et al., 2021; Jayakar et al., 2021; Mezhov et al., 2021). Neuroplastic changes are associated with brain-derived neurotrophic factor (BDNF), which affects neuronal growth and synaptic connectivity. Research indicates that the BDNF levels in participants with FMS were lower than those in healthy controls (Iannuccelli et al., 2022).

Evidence shows that tDCS can cause cerebral excitability alterations that can persist longer than the stimulation period (Farnad et al., 2021; Santos et al., 2021), offering compelling insights into its potential impact on neuroplasticity. Further substantiating this view are animal experiments, which have demonstrated that tDCS enhanced LTP, reduced LTD, and increased BDNF concentration in some areas in the brain of rats (Kronberg et al., 2017; Yu et al., 2019). Another investigation suggested that tDCS can decrease BDNF levels and decrease pain in people with knee pain, and it supported an association between change in BDNF and change in clinical pain (Suchting et al., 2021).

Transcranial direct current stimulation can induce neuroplasticity in a manner dependent on N-methyl-D-aspartate receptors (NMDARs) (Liebetanz et al., 2002; Nitsche et al., 2003), which can regulate signaling pathways by allowing positively charged ions, such as calcium, to enter the cell and strengthen the synapse. Research also discovered that tDCS increased the amount of NMDA receptors and subsequently enhanced pain-related responses in animals (Li et al., 2022a,b). This suggests tDCS enhances NMDAR-mediated synaptic plasticity by increasing neuronal membrane NMDAR density, heightening synaptic responsiveness crucial to modulating pain.

Accordingly, tDCS can trigger long-term neuroplastic changes in the brain. These changes are crucial in FMS, as they can lead to a reorganization of the pain processing pathways in the brain. Alterations in plasticity-related pathways may be accomplished by inducing LTP and upregulating BDNF or NMDARs.

## 4 Conclusion

Overall, we found that tDCS may reduce FMS pain by altering neuronal activity, regulating neuroinflammation and neurotransmission, accelerating rCBF, and inducing neuroplasticity. Deeper exploration, such as molecular studies, is needed to fill the ongoing gaps between the complex pathophysiological factors underlying FMS pain and the specific molecular changes by which tDCS reduces FMS pain., thus optimizing the efficacy of tDCS in FMS pain management. M1 and DLPFC areas in FMS sufferers are typically stimulated with 1–2 mA of tDCS for 20 min. Research on tDCS in FMS often delivers inconsistent outcomes because of different treatment protocols. This variability challenges synthesizing evidence and limits research results to broader patient populations, which underscores the need for standardized protocols to increase the comparability and generalizability of tDCS results in FMS.

While our research highlights the potential of tDCS in FMS pain relief, we need to admit that our limitations for possible biased sampling cannot be ruled out without a robust systematic literature assessment. Further research utilizing rigorous quality evaluation approaches is needed to enhance confidence in synthesizing findings. Also, this work primarily addressed immediate outcomes like pain reduction rather than long-term efficacy and impact on other symptoms. Pain location and perception vary among FMS sufferers, and they may have multi-faceted impairments beyond pain alone. It is therefore imperative that future research employs longitudinal study designs to evaluate the sustained effects of tDCS on pain symptoms and the broader spectrum of FMS manifestations. Future studies should also focus on identifying biomarkers to predict individual responses to tDCS, enhancing the treatment’s efficacy and personalization.

## Author contributions

X-QW: Conceptualization, Supervision, Writing—review and editing. SW: Methodology, Resources, Writing—original draft, Writing—review and editing. S-HD: Methodology, Resources, Writing—original draft, Writing—review and editing. J-YL: Conceptualization, Supervision, Formal analysis, Validation, Investigation, Resources, Visualization, Writing—review and editing
