# Supplementary material for: Mechanisms of transcranial direct current stimulation (tDCS) for pain in patients with fibromyalgia syndrome
Source: Front Mol Neurosci. 2024 Jan 30;17:1269636. doi: 10.3389/fnmol.2024.1269636 (PMC10865494; doi:10.3389/fnmol.2024.1269636)
Supplement: Supplementary file 1 [file Data_Sheet_1.docx]

Search strategy for PubMed：2022.11.07

#1 “Transcranial Direct Current Stimulation” [MeSH Terms] OR “Transcranial Direct Current Stimulation” [Title/Abstract] OR “tDCS” [Title/Abstract] OR “Cathodal Stimulation Transcranial Direct Current Stimulation” [Title/Abstract] OR “Cathodal Stimulation tDCS” [Title/Abstract] OR “Cathodal Stimulation tDCSs” [Title/Abstract]

**7858**

#2 “Fibromyalgia” [MeSH Terms] OR “Fibromyalgias” [MeSH Terms] OR “Fibromyalgia-Fibromyositis Syndrome” [MeSH Terms] OR “Fibromyalgia Fibromyositis Syndrome” [MeSH Terms] OR “Fibromyalgia” [Title/Abstract] OR “Fibromyalgias” [Title/Abstract] OR “Fibromyalgia-Fibromyositis Syndrome” [Title/Abstract] OR “Fibromyalgia Fibromyositis Syndrome” [Title/Abstract]

**13,459**

#3 #1 AND #2

**75**
